# Radon Concentrations in Dwellings in the Mining Area—Are There Observed Effects of the Coal Mine Closure?

**DOI:** 10.3390/ijerph19095214

**Published:** 2022-04-25

**Authors:** Małgorzata Wysocka, Sandra Nowak, Stanisław Chałupnik, Michał Bonczyk

**Affiliations:** Główny Instytut Górnictwa, Plac Gwarków 1, 40-166 Katowice, Poland; schalupnik@gig.eu (S.C.); mbonczyk@gig.eu (M.B.)

**Keywords:** abandoned coal mines, indoor radon concentration, radon migration, coal mining, doses to inhabitants

## Abstract

The article presents the results of radon research, carried out in the area of the mining commune in the Upper Silesian Coal Basin (USCB), Poland. Past investigations in the 1990s on radon concentrations in buildings, located within the mining area, showed that the indoor radon concentrations measured in the area affected by mining were higher than in buildings located outside that area. Currently, all underground hard coal mines within the boundaries of the observed commune have been closed. In 2020, after the closure of the last active mine, radon measurements were started again. The current results of indoor radon concentrations were compared with the archival results from the 1990s. It was found that the radon concentration increased significantly in the basements of buildings where measurements were made in 1990, 2020, and 2021: the maximum values were 260 Bq/m^3^, 644 Bq/m^3^, and 1041 Bq/m^3^, respectively. Therefore, these questions were posed: Do the mine closure processes increase radon migration? How long is the period of the occurrence of changes in radon concentrations in buildings after the cessation of mining operations?

## 1. Introduction

The Council Directive 2013/59/EURATOM of 5 December 2013 [1] established basic safety standards for the protection against the hazards related to ionizing radiation, which is focused particularly on issues connected with radon exposure. According to the Handbook of the World Health Organization (WHO) on indoor radon, “Since even a single alpha particle can cause major genetic damage to the cell, it is possible that radon-related DNA damage can occur at any level of an exposure” [2]. A statistically significant increase in the risk of lung cancer occurs as a result of prolonged exposure to radon. All members of the general public are exposed to radon and radon decay products. However, some populations are exposed to higher doses caused by exposure to elevated radon concentrations. Investigations, conducted all over the world, show that indoor radon concentrations can reach values that can cause a significant health risk for inhabitants [3,4,5,6,7,8,9,10,11,12].

The major source of radon lies beneath the building, in soil or rock that always contain certain concentrations of uranium and thorium, the parent radionuclides of radium isotopes (Ra-226 and Ra-228) and radon ones (Rn-222 and Rn-220), although usually only the first nuclide is recognized as radon. Soil characteristics are a very important factor determining gas movement to the surface. Radon atoms, present in that soil or rock, can enter the building through cracks in floors and walls, openings around sump pumps and drains, cavities in walls, joints in construction materials, gaps around utility penetrations, and crawl spaces that open directly into the building. Our investigations performed in the Upper Silesian Coal Basin showed that increased radon migration and its entering into buildings was caused by changes in the geological environment, due to the exploitation of mineral resources. The soil characteristics that allow gas movement to the surface in the investigated area and mining-induced changes of the rock body are among the main factors influencing radon’s ability to migrate over long distances. 

Radon emission studies in the mining areas of the Upper Silesian Coal Basin (USCB) in Poland have been carried out since the 1990s [13,14]. The analysis of the results made it possible to assess the correlation between the radon level and the geological conditions as well as the influence of historical and current mining activities. Previous measurements, performed in the area of interest, showed that radon concentrations in soil gas ranged from about 120 up to 67,000 Bq/m^3^, while radon exhalation rates varied from about 2 up to roughly 450 mBq/m^2^s. The highest values of radon exhalation rates were measured in the area of a former settling pond of coal mine waters [14,15].

The average concentration of radon in the ground floors of houses, estimated for the whole area of Upper Silesia, was 47 Bq/m^3^, while there was a higher average value for basements—64 Bq/m^3^. However, it has been indicated that a significant variation of radon levels in different zones were observed [15]. 

The relationship between the local geology and radon levels can generally be described as follows:

- Low radon potential is, in most cases, correlated with the presence of Tertiary Miocene deposits in the southern part of Upper Silesia. These sediments were formed by impermeable clays that limit gas migration;
- Elevated concentrations of radon were observed mostly in areas of the occurrence of permeable Triassic limestone and dolomite. The formations of carbonate rocks exhibit a specific geological structure—characterized by the presence of numerous cracks, many fissures, a low spatial density, and a porosity higher than that of the surrounding rocks [15,16].

In the last decade, some of the mines in the USCB were already closed or in the process of being liquidated. Observations and measurements carried out in post-mining areas are aimed at determining the potential effects and long-term threats/hazards for the inhabitants of the areas, caused by increased radon emissions, after the closure of the mines.

Within the frame of this work, we performed a series of measurements of radon concentrations in buildings, in the soil gas, and its exhalation rates in the area of one of the Upper Silesian communities. Several mines were extracting hard coal there during the last 50 years. At the beginning of 2020, the last coal mine within the border of the town was closed down. The goal of the work was to compare historical and current results of the radon levels in the context of the disintegration of the rock body, due to hard coal mining and the influence of that phenomenon on radon migration. 

## 2. Materials and Methods

### 2.1. The Research Area

**Figure 1:**
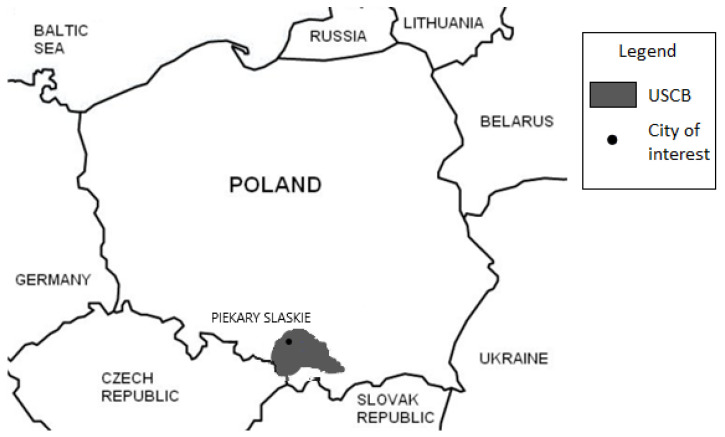
Location of the area of investigation.

Measurements were conducted inside one of the towns located in the northern part of the USCB in the Silesian Voivodship, Figure 1, within the Bytom Basin geological unit. The history of zinc–lead ore mining in this area dates back to the 12th century. During this stage of mining development, covering the period from the 12th to the 16th century, shallow deposits of galena were exploited. At that time, those parts of the outcrops that were above the water level, i.e., to a depth of several to 20 m, were selected and extracted. In the following centuries, exploitation reached deeper levels, with new shafts and other mine workings being drilled. According to the 18th-century “Memorial on the mining of ores and silver in Upper Silesia” [17], there were over 20,000 shafts in the Bytom Basin. The author mentioned that 63 shafts operated in Piekary Śląskie in 1539, and sixty years later, i.e., in 1569, there were as many as 305 shafts. With time, the drilling of galleries and chambers began at depths exceeding even 110 m. The last ore mine, called Szarlej [18], was closed in 1896. The history of coal mining in this mining commune is much shorter than that of ore mining. The first hard coal excavation was established in 1903, and four years later the mining of coal started, reaching the level of 320 metres below the surface. Another hard coal mine was established in the 1950s. Over time, coal seams from the deeper levels, 415 and 616 m, were selected for exploitation. Coal mining under the town ended in 2020.

A brief description of the history of ore and hard coal mining in the Bytom Basin area provides an idea of how many different mining excavations were conducted over several centuries in the town with an area not exceeding several dozen square kilometres. Changes in the strata, caused by the exploitation of ores and hard coal, resulted in the formation of paths for easier gas migration, including radon.

Within the frame of radon monitoring performed in the study area in the 1990s, three areas were identified:

1. areas where contemporary hard coal mining was performed under historical ore mines;
2. areas where only hard coal were mined;
3. where no mining activity was carried out.

The highest radon concentrations were measured in buildings located in the first area, while the lowest values were in the third one. In the area where no mining activity was carried out, the average radon concentration was significantly lower than in the areas affected by mining.

**Figure 2:**
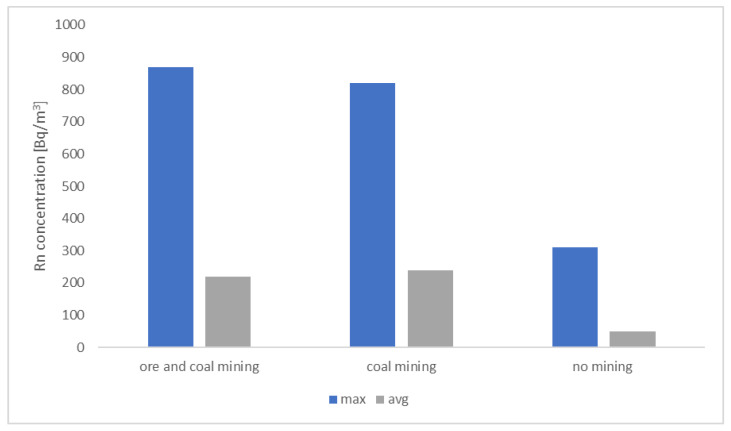
Radon level in dwellings in relation to the mining activity.

Figure 2 shows the effect of the extraction of mineral resources on the radon level in dwellings in a mining community [15]. However, extended research showed that the diversified geological structure of the area also strongly determines the migration of radon [19]. Similar problems regarding an elevated radon potential in mining areas were reported in Saar Basin, Germany, in zones where mining operations were conducted at shallow depths. They observed that elevated concentrations of radon in the soil, increasing the radon risk in dwellings, occur in a relatively narrow zone: the boundary that overlaps the area of subsidence of the surface above the underground exploitation [20,21]. In Luxembourg, changes in radon concentrations in the surface layer, which were related to mining-induced subsidence, were observed by Kies et al. [22].

### 2.2. Geology

The Upper Silesian Coal Basin was formed during the Variscan orogeny and rejuvenated during the Alpine [23,24]. The geological structure of the Basin is complicated and varied, with the rock body additionally being strongly tectonised and cracked by faults. In the geological structure of the Piekary Śląskie site, the following geological formation could be distinguished:

- Quaternary (Holocene, Pleistocene) built of clays and sands;
- outcrops of Triassic deposits;
- outcrops of Coal-Bearing Carboniferous, the basic coal deposit including the Upper Silesian sandstone series, and mudstone series.

The Coal-Bearing Carboniferous, a typical multi-facies formation, is composed of clastic rocks and coal seams. The lack of limestone is a characteristic feature of this association. The Carboniferous strata are usually overlain by younger deposits, ranging from Triassic to Quaternary. Triassic strata are of an erosion character. These deposits occur in the form of isolated remnants from larger entities. The younger Quaternary series, deposited under continental conditions, is undisturbed by faults and occurs as continuous layers [25].

The Triassic rocks are represented by Muschelkalk ore-bearing and diploporita dolomites. These sediments are characterized by high fracturing with many fissures, a small spatial density, and a porosity higher than that of the surrounding rocks [26]. Carbonate rocks occurring in the research area are characterized by intense fractures, and the presence of numerous fissures and voids. The physical characteristics of the bedrock described above facilitate gas migration. The Quaternary deposit of different thicknesses is built of multi-grained sands and gravel, as well as dust, clays, and loams. 

## 3. Methods Applied

### Track Detectors

**Figure 3:**
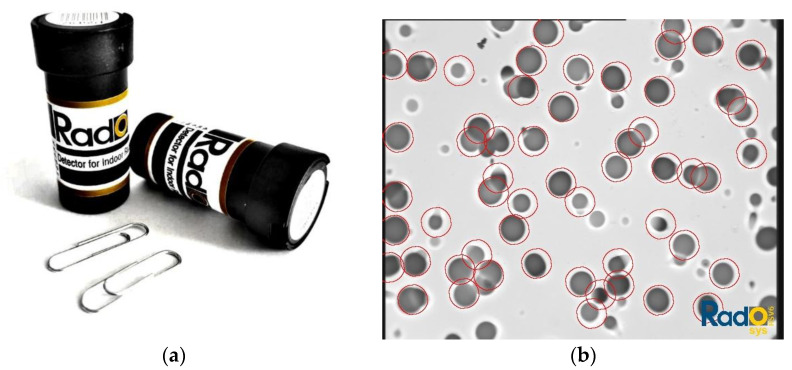
(**a**) Solid-state track detector RadoSys used in our work; (**b**) CR-39 foil (an example of damages after exposition).

In our study, measurements of ^222^Rn concentrations in indoor air were carried out using solid-state nuclear track detectors. The detectors, from the Hungarian company RadoSys, series Z and AL, consist of a diffusive chamber with CR-39 foil. An exemplary track detector is depicted in Figure 3a.

CR-39 foil is a plastic polymer, sensitive to alpha particles emitted by radon and its progeny. Alpha particles destroy chemical bonds in the polymer structure, producing microscopic defects in the material, called tracks, Figure 3b. The number of tracks was counted with an optical microscope. Radon concentration is directly related to the number of tracks in the CR-39 foil. Detectors were exposed for at least 3 months in each house in the basement and in a room on the ground floor. The applied method enables the lower limit of detection 8 Bq/m^3^ to be reached for a 3-month measurement period. 

For the estimation of the uncertainty of the calculated radon concentration, following factors should be taken into consideration:

- the uncertainty of the track density, readout done by the optical microscope type RADOMETER MICROSCOPE with appropriate software;
- the uncertainty of the calibration factor, applied, accordingly to the producer, as 5% of the calibration factor value, expressed in ((mm^2^/track)·(Bq·h/m^3^));
- the uncertainty of the exposure time—6 h;
- the uncertainty of the track background density equal 0.1 track/mm^2^.

The overall uncertainty U(CRn) of the calculated radon concentration in air is given as an expanded uncertainty with the coverage factor k = 2 (with a probability of about 95%).

The value of the uncertainty is approximately 10% of the calculated value of the radon concentration.

The CR-39 detectors that were used in our work are placed in low diffusion chambers to prevent the entry of thoron into it. According to information of the producer, RadoSys company, thoron influence on radon results is negligible. RadoSys offers a special construction of dual chambers, one of which is low diffusion and second one is high diffusion to measure simultaneously radon and thoron levels, but in presented work these types of detectors were not used. 

CR-39 detectors were installed on two levels in each monitored building. In case of residential houses, we put 2 detectors at one measuring point in basements at the distance of about 1 m from the walls and 1.5 m from the floors. Another two detectors were put at one point in bedrooms according to the scheme used in the basement. In public utility buildings, as in residential houses, 2 detectors were installed in the basements and in the rooms on the ground floor. The rooms on the ground floors were indicated by users. In schools and kindergartens, these were classes or offices of managers. In the hospital and in the buildings of the city hall, these were offices where employees work for about 8 h daily. 

The total number of monitored buildings were 56. In eight of these buildings, the first measurement campaign was conducted in 1990 and repeated in 2020 and 2021. Measurements continue in all 56 buildings.

In order to obtain knowledge about the doses to which city residents may be exposed as a result of radon exposure, annual effective doses for the highest radon concentrations could be calculated based on the UNSCEAR model:

E_ainh_ = DCF_Rninh_ · C_Rna_ · t_r_ · F

where:

E_ainh_—effective dose from inhalation of indoor air radon [mSv/year]
DCF—the dose conversion factor 1.4 × 10^−8^ Sv/(Bq/m^3^·h) were taken from report ICRP (2017).
C_Rna_—indoor air radon concentration [Bq/m^3^]
t_r_—hours spend by man in house [h]
F—the equilibrium factor between radon and progeny—the value is set as 0.4

Usually, as an average number of hours spend by people in house, 7000 h/year is adopted. In our dose evaluation we used 7000 h/year for each place in house.

## 4. Results 

During the first campaign, solid-state track detectors were exposed within the period 10 October 2020 to 4 April 2021 in 21 buildings in the study area. Radon measurements were taken in basements and on ground floors. In eight of them, measurements were performed in exactly the same buildings monitored in the 1990s. 

The radon concentrations in buildings measured in the years 2020–2021 range from the lower limit of the currently used method, i.e., 8 Bq/m^3^, to 1886 Bq/m^3^, with the arithmetic average 111 Bq/m^3^ and median 52 Bq/m^3^. Higher concentrations were measured in the cellars, up to 1886 Bq/m^3^, with the arithmetic average 159 Bq/m^3^ and median 142 Bq/m^3^.

**Figure 4:**
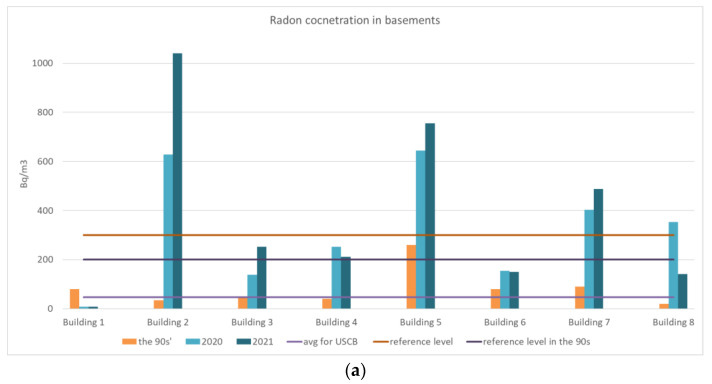

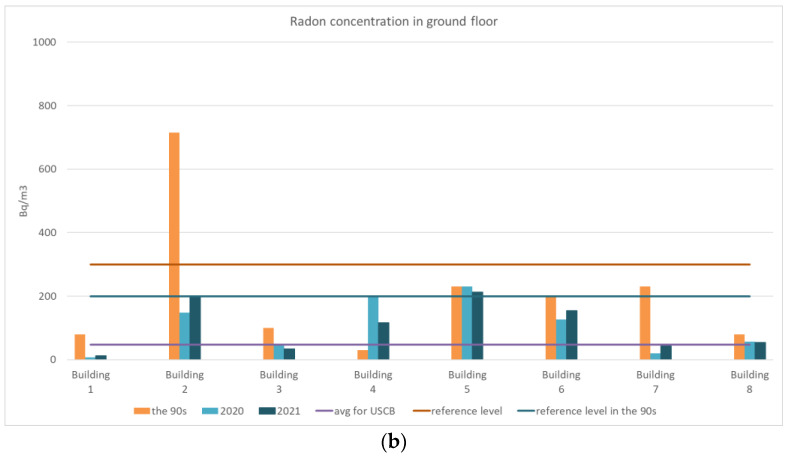
Radon concentrations, measured in the (**a**) basements and (**b**) on the ground floors of the same buildings in the 1990s and in the years 2020–2021.

The results obtained within the frame of the last campaign were compared with the data collected in the 1990s for basements and ground floors in Figure 4a,b, respectively. In general, it can be observed that both maximum and average values calculated based on the results of the years 2020 and 2021 are higher than in the 1990s. However, the changes of radon concentrations in basements and on the ground floor show clearly the different trends. The highest measured value of radon concentrations measured during the last campaign (the year 2021) reached 1886 Bq/m^3^, while the highest value measured in the last century reached 860 Bq/m^3^. On the contrary, we observe a decrease in radon levels, measured on ground floors in all buildings except one.

**Figure 5:**
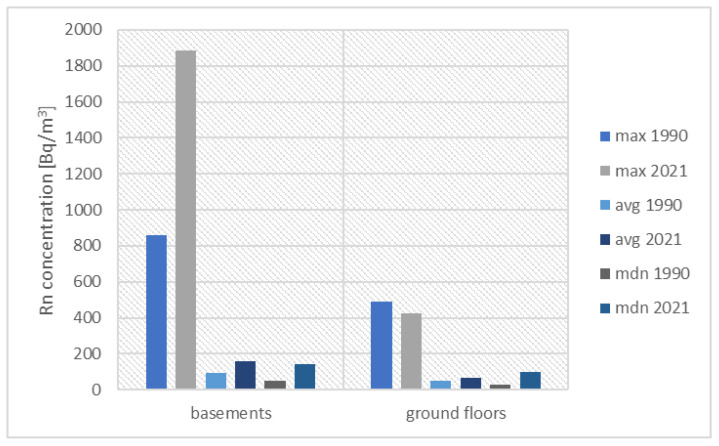
Comparison of radon concentrations in buildings in Piekary Śląskie measured in the 1990s and nowadays.

In 2021, the measurements were extended by another 35 buildings, mainly public buildings, such as schools, kindergartens, hospitals, and municipal offices. The buildings with which the measurement base was extended are located in the vicinity of the buildings tested in the last century. The first series of measurements in the expanded number of buildings was carried out in the spring–summer 2021 period. The results of measurements carried out in all buildings monitored in the last century and currently monitored were compared. In the currently monitored houses in the basement, clearly higher values were measured compared to the archival data (860 Bq/m^3^ vs. 1886 Bq/m^3^). On the other hand, in the rooms on the ground floor, the average radon concentration is only slightly higher nowadays than that calculated on the basis of archival data (50 Bq/m^3^ vs. 64 Bq/m^3^), see Figure 5.

For a deeper analysis of the results of the comparison of “old” and “new” data, the locations of the buildings in which the research was conducted were divided due to the geological structure of the underlying strata:

- outcrops of the Coal-Bearing Carboniferous;
- Triassic outcrops;
- a layer of Quaternary sediments, over 10 m thick.

**Figure 6:**
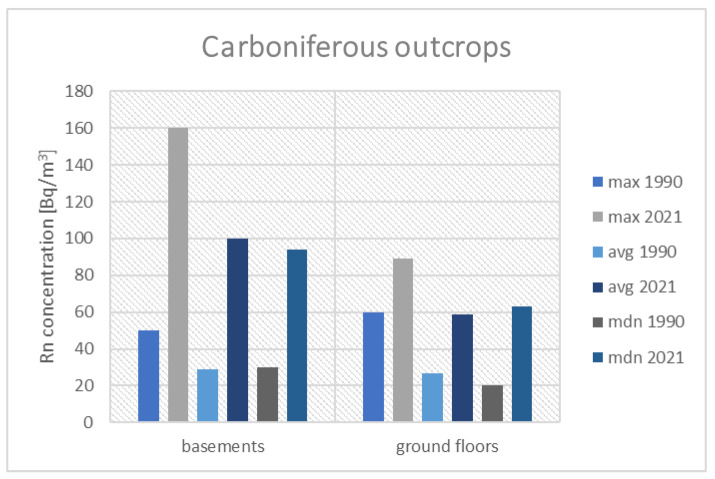
Comparison of radon concentrations in buildings located on Carboniferous outcrops.

**Figure 7:**
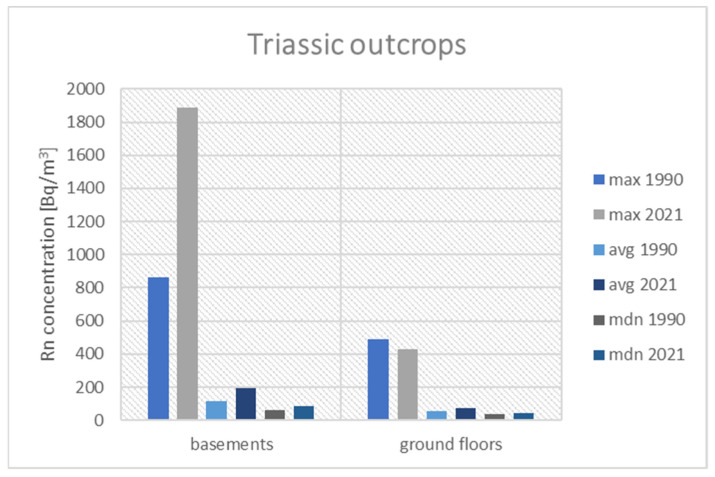
Comparison of radon concentrations in buildings located on Triassic outcrops.

**Figure 8:**
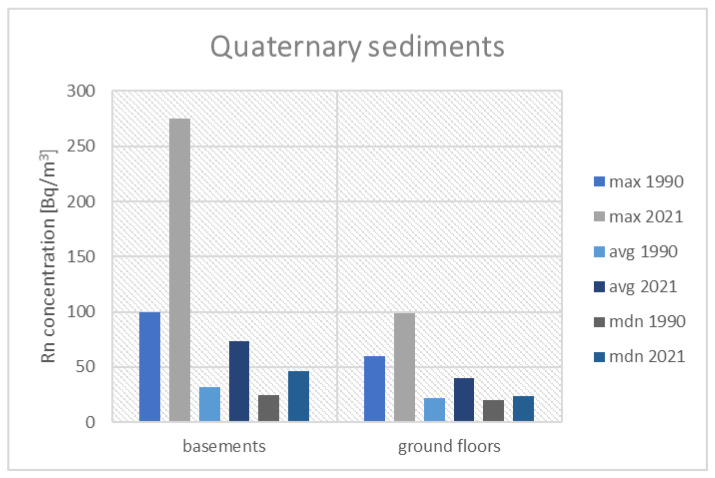
Comparison of radon concentrations in buildings located on Quaternary sediments.

Figure 6 presents the results of the historical and current measurements performed in buildings built on Coal-Bearing Carboniferous outcrops. The following figures (Figure 7 and Figure 8) present the results for a group of houses located on the Triassic outcrop and built on Quaternary sediments.

In buildings built on Carboniferous deposits, in 2021 we measured higher radon concentrations than in the last century, both in the basements and on the ground floors. At both levels, the mean values (29 Bq/m^3^ vs. 100 Bq/m^3^ in the basements and 27 Bq/m^3^ vs. 59 Bq/m^3^ on the ground floors) and the medians (30 Bq/m^3^ vs. 94 Bq/m^3^ in the basements and 20 Bq/m^3^ vs. Bq/m^3^ on the ground floors) are significantly higher than the values measured in the past. However, these are values below the reference value of 300 Bq/m^3^.

In buildings built on Triassic carbonate formations, a significant increase in radon concentrations in the basements was noticed. Both the maximum value of radon concentrations measured in 2021 and the calculated average are higher than the archival values (1886 Bq/m^3^ vs. 860 Bq/m^3^). The median of 85 Bq/m^3^ and mean of 193 Bq/m^3^ are also higher than the values from the 1990s, which were 60 Bq/m^3^ and 114 Bq/m^3^, respectively. In rooms on the ground floor, the maximum measured value of radon concentration (426 Bq/m^3^) is currently slightly lower than that measured 30 years ago (490 Bq/m^3^). The values of the mean and median, calculated on the basis of contemporary measurements are similar to the archival values (74 Bq/m^3^ vs. 56 Bq/m^3^ and 43 Bq/m^3^ vs. 51 Bq/m^3^). It can therefore be assumed that the radon level on the ground floors has been stable for over 30 years. However, it should be emphasized that in this location in buildings, not yet monitored, the radon concentrations may exceed the reference level.

In basements in buildings, built on Quaternary sediments, the maximum concentrations of radon, measured during the measurement campaign of 2021 (275 Bq/m^3^), the average value (73 Bq/m^3^) and the median (46 Bq/m^3^) are higher than in the past (100 Bq/m^3^, 32 Bq/m^3^, and 25 Bq/m^3^). The situation is similar in the case of rooms on the ground floor; max 99 Bq/m^3^ vs. 60 Bq/m^3^, avg. 40 Bq/m^3^ vs. 22 Bq/m^3^, and median 24 Bq/m^3^ vs. 20 Bq/m^3^. Despite the observed increase in radon concentrations in the basements and on the ground floors, we do not expect the reference value to be exceeded in this location.

## 5. Discussion

Archival results of radon concentrations from the 1990s were compared with the results of monitoring performed in 2020–2021. When analyzing all the results of current measurements, it has been found that the values of radon concentrations in basements, measured nowadays, are higher than the values measured in the previous century. Therefore, it can be assumed that the physical parameters of the rock mass below the building facilitate gas migration to a greater degree now than in the last century. On the ground floors, radon maximum concentrations are slightly lower than in previous years, while average and median values are higher. It means that the distribution of the currently measured concentration is less dispersed in comparison to historical data. It confirms that even in ground floors, we measure higher radon concentrations. The radon penetration into the ground floor rooms is limited, especially in buildings that have been renovated in recent years due to damages, caused by mining. Often the cause of damages to buildings was the subsidence of the surface above the voids, left after the exploitation of mineral resources. The distribution of radon concentrations was analyzed against the background of the geological structure of the monitored area. An increase in radon concentrations in basements was found in buildings built on any type of geological litho-stratigraphic units, i.e., on Triassic carbonate rocks, Carboniferous shales and sandstones, as well as Quaternary sediments. The highest measured values of radon concentrations and the highest average values are found in houses located on Triassic outcrops. In the case of this geological formation, the dynamics of the increase in radon concentrations are the most visible in basements. On the ground floors, the currently measured concentrations are comparable with the archival values. Both in the basements and rooms on the ground floor, values above the reference value of 300 Bq/m^3^ were measured. In buildings located on Carboniferous outcrops and Quaternary sediments, significant increases in radon concentrations are visible both in the basements and on the ground floors. Despite the observed dynamics of the increase in concentrations, no values above the reference value were measured. 

The impact effects of centuries of mining activity on gas migration in the subsurface layer were analyzed. In the area covered by measurements, zinc and lead ores were mined since medieval times, and, starting from the beginning of the 20th century, also hard coal exploitation started there. The last hard coal mine ceased its operations in 2020. According to the information obtained from mining specialists, at present, there are no longer any effects of mining on surface deformation, or they are of a residual nature. However, the disintegration and changes in the strata, caused by centuries of mining activities are irreversible. Physical and chemical processes occurring in the subsurface layer and at deeper levels may influence the migration of fluids. The dynamics of gas migration are determined by many factors and processes, directly or indirectly related to the effects of underground mining:

- Surface erosion, causing the disintegration of rock material, usually reaches 15 m. In Triassic limestone and dolomite, disturbed by the exploitation of mineral resources, the erosion takes place at much greater depths, even up to 100 m;
- as a result of stresses caused by mining activities, some of the tectonic faults are rejuvenated and sometimes reopened, clogged faults are usually accompanied by a zone of contemporary cracks, creating the pathways for gas migration;
- post-mining areas are often drained, and the groundwater level is lowered, which enables the easier migration of fluids;
- deep mining exploitation may cause surface discontinuities—driving excavation in the vicinity of a fault zone even at a depth of 800 m may result in surface damages.

Only a very rough estimation of doses for inhabitants has been done—estimated doses for residents in the rooms on the ground floor range from 0.1 to about 7.0 mSv/year. The work is in progress and after completion, the doses will be calculated based on the results of the year-long radon concentration measurements. An analysis of the distribution of radon concentrations in buildings will be performed, taking into account the technical parameters of buildings, such as type, age, construction and finishing materials, renovations after damages, etc. 

## 6. Conclusions

The analysis of the results of measurements, conducted in Piekary Śląskie, confirmed the hypothesis about the primary influence of the local geological structure on the distribution of radon levels in buildings. The exploitation/extraction of coal beds causes the fracturing of rigid layers of sandstones and shales lying above. In the area of Piekary Śląskie, above the Carboniferous rocks lie Triassic carbonate formations (limestones and dolomites), in which metal ores have been exploited since the Middle Ages. When we started our work, we assumed that we would measure the highest radon concentrations in buildings located over exploited coal and metal ore deposits. In fact, the highest radon concentrations in basements were measured in buildings situated on Triassic dolomites, above Carboniferous formations. Mineral resources were exploited within both of these formations. However, another interesting regularity was observed: in basements of buildings located on each of the geological formations occurring in the study area, radon concentrations measured today are higher than the values measured in the 1990s. In no case are they as high as the maximum concentrations in basements of buildings situated on Triassic dolomites and limestones.

Thus, it may be assumed that changes in the rock mass, result from mining occurrence in each geological formation. These changes are accelerating the phenomena of surface erosion and karst development (presence of voids, subsidence, lowering of the water level, rejuvenation of faults, etc.). It leads to the creation of pathways for more intense gas migration. However, in Triassic carbonate rocks, mentioned above, such phenomena are the most intensive and developed. Moreover, it should be assumed, that in post-mining regions, areas with an increased radon risk occur not only directly above mining excavations or subsidence zones, but also in other zones where rock mass disintegration leads to erosion and karst formation. 

Therefore, our work will be continued, and the most important challenges are to determine (together with mining experts) the real boundaries of subsidence basins formed as a result of mining, to compare the rate and volume of subsidence in the 1990s and in the 2000s, and to collect and compare data on changes in groundwater levels and the migration of other gases, such as methane. These factors can affect the migration of gases in the rock mass.

## Data Availability

Analyses in this study were based on existing data of radon concentration in dwellings. The measurements were performed by the authors of the paper and gathered in our database. Data sharing is not applicable to this article.
